# Evaluating outcomes of patient-centered enhanced recovery after surgery (ERAS) in percutaneous nephrolithotomy for staghorn stones: An initial experience

**DOI:** 10.3389/fsurg.2023.1138814

**Published:** 2023-03-21

**Authors:** Jun Lei, Kai Huang, Yingbo Dai, Guangming Yin

**Affiliations:** ^1^ Department of Urology, Third Xiangya Hospital, Central South University, Changsha, China; ^2^ Department of Urology, The Fifth Affiliated Hospital of Sun Yat-sen University, Zhuhai, China

**Keywords:** patient-centered, eras, PCNL, staghorn, recurrence

## Abstract

**Objective:**

To evaluate the outcomes of patient-centered enhanced recovery after surgery (ERAS) in ­percutaneous nephrolithotomy (PCNL) for staghorn stones.

**Patients and methods:**

A retrospective analysis of 106 patients with staghorn calculi who underwent PCNL treatment at the Third Xiangya Hospital from October 01, 2018 to September 30, 2021 was performed. The patients were divided into the ERAS group (*n* = 56) and traditional group (*n* = 50). The ERAS program focused on a patient-centered concept, with elaboration on aspects, such as patient education, nutritional support, analgesia, body warming, early mobilization, nephrostomy tube removal, and strict follow-up.

**Results:**

The total stone free rate and total complication rate were similar in both groups. The visual analogue scale (VAS) 6 h after surgery, ambulation off bed time, indwelling ﬁstula time, indwelling catheter time, and postoperative hospital stays were lower in the ERAS group than in the traditional group (*P* < 0.05). The multiple session rate in the ERAS group (19, 28.57%) was lower than that in the traditional group (30, 60%) (*P* = 0.007). The 1-year stone recurrence rate in the ERAS group (7, 17.5%) was lower than that in the traditional group (14, 38.9%) (*P* = 0.037).

**Conclusion:**

The patient-centered ERAS in PCNL for staghorn stones accelerated rehabilitation by relieving postoperative pain, shortening hospitalization time, accelerating early ambulation, and reducing multiple session rate and 1-year stone recurrence rate, which have socioeconomic benefits.

## 1. Introduction

Staghorn calculi are large kidney stones located in the renal pelvis and extending into calices. If untreated, they may cause renal failure and even life-threatening urosepsis with time (1–3). Although the proportion of Staghorn calculi among all urinary stones has been reduced to 4% due to early and effective management of renal stones in developed countries, incidence remains 10%–20% in developing countries (4, 5). Among all treatment options for staghorn calculi, percutaneous nephrolithotomy (PCNL) is recommended as the gold standard (6, 7). However, PCNL is often performed in several stages with potential of major postoperative complications, including severe bleeding and urosepsis, occurring at a rate of 1.1%–7.0% (8, 9). Furthermore, patients with Staghorn calculi often have poor economic backgrounds and possible underlying infections and malnutrition (5, 10, 11).

First applied in Denmark by Kehlet (12), enhanced recovery after surgery (ERAS) was introduced in China by Jiang (13) in 2007. ERAS refers to the optimization of the clinical pathway during perioperative management based on evidence-based medicine practiced by multi-department collaboration in surgery, anesthesia, and nursing (12, 14). It can significantly alleviate perioperative stress response and complications, shorten hospital stay, and accelerate rehabilitation in several surgical specialties (15–18). However, the literature on the suitability of the ERAS program for use of PCNL in treating Staghorn calculi remains limited.

Therefore, to determine whether patients receiving PCNL for staghorn stones could also benefit from the ERAS protocol, this study systematically investigated the evidence and guidelines published in English and Chinese databases, developed a patient-centered ERAS protocol, and evaluated the outcomes.

## 2. Materials and methods

### 2.1. Establishment of ERAS protocol

A comprehensive literature review was performed on literature available in databases, such as EBSCO Medline, PubMed, Elsevier, NGC, CNKI, and Wanfang. After screening using keywords, such as “enhanced recovery after surgery,” “fast track surgery,” “kidney stone,” and “ Staghorn calculi,” one Chinese expert consensus (19), nine clinical practice guidelines (20–28), three reviews (11, 29, 30), and one randomized controlled trial (RCT) (31) were selected for developing the ERAS protocol. It is detailed in Table 1.

**Table 1:** Detailed patient-centered ERAS program.

Management	ERAS group (*n *= 56)	Traditional group (*n *= 50)
**Preoperative**		
1. Education	Detailed information, including ERAS concept, PCNL advantages and disadvantages, perioperative complications and corresponding treatment measures, and importance of cooperation	Information about PCNL surgery
2. Training	Practicing abdominal breathing, regulating respiratory frequency, lying prone above a cushion, exercising lower limbs on the bed	Train lower limbs exercise on the bed.
3. Nutrition	Nutritionist consultation and building personalized nutrient program	Conventional nutrition intake
4. Bowel preparation	No bowel preparation except for constipation	Lactulose 40 ml mixed with 1 L of warm water
5. Fasting	No solid food intake for 6 h before surgery. 400 ml 5% glucose drink 2 h before surgery. Xylitol, if patient has diabetes	No food intake for 12 h before surgery. No liquid intake for 8 h before surgery.
6. Psychological care	Show patients a successful endoscopy video of the lithotripsy procedure. Ask for their permission to share the lithotripsy procedure with themselves and other patients.	No
Intraoperative		
1. Anesthesia	General anesthesia or continuous epidural anesthesia after anesthetist consultation	Same as the ERAS group
2. Nutrition	According to personalized nutrient program, goal-directed fluid therapy for intraoperative fluid administration. The standard ranges from 10 ml/kg to 15 ml/kg body weight.	500–1,000 ml of crystalloid solution
3. Medicine	Parecoxib 40 mg or ﬂurbiprofen 50 mg infusion and dexamethasone 10 mg infusion for reducing postoperative wound inﬂammation Silansetron 5 mg or granisetron 50 ml sodium chloride intravenous infusions to prevent vomiting after surgery Culture-specific antibiotics or broad-spectrum antibiotics 30 min before surgery	Culture-specific antibiotics or broad-spectrum antibiotics 30 min before surgery
4. Warming	Strengthen monitoring the body temperature. Keep the operating room temperature 24–26°C. Warm intravenous fluids and surgical infusion fluids for lithotripsy.	Keep the operating room temperature at 24–26°C.
5. Communication	Share a live video of lithotripsy on a small screen, if patients permitted before surgery. Ask for patient's coordination on respiratory frequency during lithotripsy, if patient is awakened.	No communication
Postoperative		
1. Analgesia	Preemptive analgesia during operation. Multimodal analgesia based on NSAIDs, including parecoxib 40 mg intramuscular injection or ﬂurbiprofen 50 mg intravenous drip, when patient returns to the ward. Personalized administration based on VAS scoring 6 h after surgery. Diclofenac sodium 2 ml intramuscular injection, if VAS >4	Give a VAS scoring 6 h after surgery Diclofenac sodium 2 ml intramuscular injection, if pain is unbearable Analgesic pump on demand
2. Vomiting prevention	Preventive use of antiemetics at the end of surgery. Metoclopramide and serotonin (5-HT3)-receptor blocker on demand. Liquid diet 6 h after surgery.	Metoclopramide and serotonin (5-HT3)-receptor blocker on demand
3. Nutrition	Chew gum.	Liquid diet after the ﬁrst flatus. 20 ml of Simo decoction (Oral liquid of Chinese medicine) to prevent constipation.
4. Mobilization	5% glucose 10 ml (xylitol for patients with diabetes) oral intake per hour. Other contents of the personalized nutrient program 20 ml of Simo decoction (Oral liquid of Chinese medicine) to prevent constipation Encourage bed exercises, such as lower limb exercises assisted by caregiver, when patient returns to the ward. Gradually transit to voluntary activities 2 h after surgery. Encourage gradual increase in activities based on the patient's situation.	Absolute bed rest for 3–4 days; encourage bed exercises.
5. Education	A paper listing the relationships between common foods and fluids and stone formation. Speciﬁc nutritional therapy based on dietary assessment and metabolic evaluation according to stone composition of every patient (Table 7).	Same as the ERAS group
Follow-up	Telephone or/and WeChat follow-up every month to monitor adherence to speciﬁc nutritional therapy. KUB or CT examination every 6 months	KUB or CT examination every 6 months

ERAS, enhanced recovery after surgery; PCNL, percutaneous nephrolithotomy; NSAIDs, nonsteroidal anti-inflammatory drugs; VAS, visual analogue scale; KUB, kidney-ureter-bladder; CT, computed tomography.

### 2.2. Patients

This study was approved by the Institutional Review Board of The Third Xiangya Hospital of Central South University (ID: fast I 21075). All patients with staghorn stones were informed whether to accept the ERAS protocol since October 2019. If accepted, they and their family members provided written informed consent for participation and the use of their clinical data in publications and were assigned to the ERAS group, which was administered by a dedicated team consisting of 4 urologists and 5 nurses who received professional ERAS protocol training. If patients and their family members did not agree, they were assigned to the traditional group. Because the majority of patients chose to receive the ERAS protocol, patients in the traditional group were insufficient after the ERAS protocol was carried out. Considering that there was no difference between the treatments of patients in the traditional group and the previous patients with staghorn stones, we selected October 2018 as the enrollment time point of the traditional group.

The inclusion criteria were (1) diagnosis of staghorn calculi by abdominal plain radiography (KUB), intravenous urography (IVU), or non-contrast computed tomography (CT); (2) age between 18 and 70 years; (3) planned initial therapy with PCNL; (4) ASA grade of I–III; (5) diabetes-controlled postprandial blood glucose ≤11.1 mmol/L, hypertension-controlled blood pressure ≤140/100 mmHg; and (6) absence of uncontrolled renal and cardiopulmonary insufficiency. The exclusion criteria were (1) in company with renal tumor, tuberculosis, and other urinary diseases; (2) presence of horseshoe kidney, ectopic kidney, isolated kidney, ureteropelvic junction obstruction (UPJO), scoliosis, and other severe malformations; (3) history of ipsilateral open surgery; (4) urosepsis requiring emergency surgery management; and (5) anticoagulant use in the past two weeks.

Thereafter, a retrospective analysis of 106 patients with staghorn calculi who underwent PCNL treatment in The Third Xiangya Hospital from October 2018 to September 2021 was performed. The patients were categorized by use of ERAS management (*n* = 56) or conventional management (*n* = 50). The detailed distinctions between both groups are shown in Table 1.

### 2.3. Common procedures

#### 2.3.1. Preoperative procedures

All patients provided detailed medical histories Preoperative examinations included routine blood tests, serum creatinine, coagulation function test, urine culture, and radiological evaluations, such as KUB, IVU and CT (mandatory), and/or urinary ultrasonography. Stone surface area was assessed as stone burden as determined by radiological examinations, such as the KUB or CT. For all patients, infections were treated and antibiotics were administered as prophylaxis preoperatively.

#### 2.3.2. Intraoperative procedures

After anesthesia, patients were placed in the lithotomy position to complete ureteral catheterization. Thereafter, they were placed in a prone position to establish PCNL tracts. Guided by ultrasound, an experienced urologist obtained All PCNL accesses. After placing the safety wire, the tract was dilated with an 8-Fr renal sheath to 20/24 Fr. Multiple tracks could be obtained, if devised in the preoperative plan; otherwise, multiple tracks could be accessed during surgery on demand. Ultrasonic pneumatic devices or holmium laser lithotripter was used to fragment stones using 18 Fr or 20 Fr rigid ureteroscope. A nephrostomy tube and double-J internal ureteral stent were routinely inserted at the end of the ﬁrst session of the PCNL.

#### 2.3.3. Postoperative procedures

Hematologic examinations, including routine blood tests, serum creatinine, and hepatorenal function, were performed immediately after patients returned from the ward. A visual analogue scale (VAS) was used to evaluate postoperative pain 6 h after surgery. Stones collected during operation were analyzed by infrared spectroscopy to confirm the chemical composition. Culture-specific antibiotics were administered, if preoperative urine cultures were positive, whereas broad-spectrum antibiotics were administered, if preoperative urine cultures were negative for 5–7 days. KUB or non-contrast CT (suggested) was ordered at least a day before discharge to assess the state of stone clearance and ascertain the double-J stent in the right position. Removal of the nephrostomy tube was based on whether the drainage had become roughly clear. If a secondary PCNL session was planned when the status of stone clearance had not been clarified, it was retained. All patients were followed up by KUB or CT (suggested) every 6 months. The internal double-J stent was removed a month later. The results of the examination during follow-up determined whether stones recurred a year after surgery.

### 2.4. Indicators

General characteristics, including age, sex, body mass index (BMI), American Society of Anesthesiologists (ASA) classification, stone size, staghorn calculi type, stone burden, distance of tract, and stone CT value, were used to evaluate baseline characteristics. Stone burden, tract length, and CT value were obtained from measurements on CT by two different urologists. The following formula was used to calculate the stone burden: length × width × π × 0.25 (32). Residual stone fragments smaller than 4 mm in diameter, which did not require surgical intervention, were counted in clearance.

Postoperative clinical data, such as operative time, multi-tract rate, hemoglobin decrease, multiple session rate, total stone free rate, and 1-year recurrence rate, were recorded. Additionally, recovery data, such as VAS 6 h after surgery, ambulation off bed time, indwelling ﬁstula time, indwelling catheter time, hospital stay, and complications were subsequently recorded for comparison between both groups.

### 2.5. Statistical analyses

IBM SPSS version 26.0 software (IBM Corp., Armonk, NY, USA) was used for all statistical analyses. Measurement data were expressed as mean ± standard deviation (SD). Two independent sample t-test was used to compare the differences between the groups. Categorical data were expressed as frequencies and percentages. The Chi-square test (*χ*2) was applied to compare the differences between both groups. *P* < 0.05 indicated that the differences between both groups had statistical significance.

## 3. Results

The preoperative characteristics of patients, including age, BMI, and stone CT value, were not different between both groups (*P* > 0.05) (Table 2). The postoperative surgical parameters of both groups are listed in Table 3. All patients successfully underwent PCNL. No intraoperative conversion to open surgery was required. No differences in operative time, tract size, tract number, multi-tract rate, hemoglobin decrease, and total stone free rate between both groups were recorded. However, the multiple session rate of the ERAS group (19, 28.57%) was lower than that of the traditional group (30, 60%) (*P* = 0.007). Additionally, the 1-year recurrence rate in the ERAS group (7, 17.5%) was lower than that in the traditional group (14, 38.9%) (*P* = 0.037). Table 4 shows the postoperative recovery parameters. No differences in hematuria duration and total hospital stay were between both groups were recorded. However, VAS 6 h after surgery (1.32 ± 0.66 in the ERAS group; 2.24 ± 0.82 in the traditional group), ambulation off bed time (1.64 ± 0.61days in the ERAS group; 2.86 ± 0.67 days in the traditional group), indwelling ﬁstula time (3.14 ± 2.54 days in the ERAS group; 4.82 ± 3.27 days in the traditional group), indwelling catheter time (2.37 ± 1.13 days in the ERAS group; 4.98 ± 1.47 days in the traditional group), and postoperative hospital stay (6.67 ± 2.09 days in the ERAS group; 7.96 ± 2.02 days in the traditional group) were different (*P* < 0.05) between both groups. Generally, these indicators were lower in the ERAS group than in the traditional group. The postoperative complications of both groups are listed in Table 5. Additionally, seven cases of fever were recorded in the ERAS group. Among these patients, one, four, and two cases were associated with urosepsis, prolonged hematuria, and transfusion, respectively. One patient with severe hematuria underwent digital subtraction angiography (DSA) embolization. Furthermore, one patient was diagnosed with hydrothorax without surgical intervention 2 days after operation. In the traditional group, six and seven cases of fever and prolonged hematuria, respectively, were recorded. Four cases were associated with transfusion, whereas one patient with hematuria underwent DSA embolization. In both groups, all complications were treated medically. No ICU admission or deaths were recorded. In Table 6, the stone composition of both groups are listed. After analyses by infrared spectroscopy, most stones were mixed stones. In this study, the stones were mainly composed of calcium, uric acid, struvite, and cystine. In the ERAS group, 37 calcium-based, six uric acid, and 13 struvite stones were identified; whereas in the traditional group, 31 calcium-based, two uric acid, one cystine, and 16 struvite stones were identified.

**Table 2:** Preoperative characteristics of patients.

Parameters^a^	ERAS group (*n* = 56)	Traditional group (*n* = 50)	*z*/*t*/*χ*^2^^b^	*P*
Age (year)	51.19 ± 12.30	49.86 ± 10.73	0.592	0.555
BMI (kg/m^2^)	23.62 ± 3.52	22.48 ± 3.45	1.675	0.097
ASA classification (*n*)			1.521	0.467
I	43	33		
II	9	12		
III	4	5		
Gender, (female, *n*, %)	25, 45%	23, 46%	0.020	0.889
Stone side (left, *n*, %)	28, 50%	21, 42%	0.680	0.410
Complete staghorn (*n*, %)	36, 64.28%	27, 54%	1.159	0.282
Stone burden (mm^2^)	1,271.63 ± 813.19	1,343.87 ± 887.29	−0.437	0.663
Distance of tract (mm)	54.79 ± 10.46	51.65 ± 9.63	1.601	0.112
Stone CT value (Hu)	975.41 ± 242.17	1,055.66 ± 175.85	−1.966	0.052

^a^ Values are expressed as mean ± standard deviation.

^b^ The independent samples *t*-test or Mann-Whitney test for continuous variables and Chi-square test for categorical variables.

BMI, body mass index; ASA, American Society of Anesthesiologists classification; CT, computed tomography; Hu, Hounsfield unit.

**Table 3:** Postoperative surgical parameters.

Parameters^a^	ERAS group (*n* = 56)	Traditional group (*n* = 50)	*z*/*t*/χ^2^^b^	*P*
Operative time (min)	120.23 ± 33.70	122.08 ± 37.47	−0.267	0.790
Size of tract (Fr)	21.35 ± 1.62	21.60 ± 1.66	−0.760	0.449
Number of tracts (*n*)	1.35 ± 0.64	1.30 ± 0.54	0. 490	0.625
Multi-tract rate (*n*, %)	16, 28.57%	13, 26%	0.088	0.767
Decrease of hemoglobin (g/L)	19.78 ± 14.06	18.34 ± 10.43	0.595	0.553
**Multiple session rate (*n*, %)**	**19, 28.57%**	**30, 60%**	7.223	**0**.**007**
Total stone free rate (*n*, %)	40, 71.42%	36, 72%	0.004	0.948
**1-year recurrence rate (*n*, %)**	**7, 17.5%**	**14, 38.9%**	4.335	**0**.**037**

^a^ Values are expressed as mean ± standard deviation.

^b^ The independent samples *t*-test or Mann-Whitney test for continuous variables and Chi-square test for categorical variables.

Fr, French.

**Table 4:** Postoperative recovery parameters.

Parameters^a^	ERAS group (*n* = 56)	Traditional group (*n* = 50)	*z*/*t*^b^	*P*
**VAS 6 h after surgery (*n*)**	1.32 ± 0.66	2.24 ± 0.82	−6.358	**0**.**000**
**Ambulation off bed time (day)**	1.64 ± 0.61	2.86 ± 0.67	−9.743	**0**.**000**
**Indwelling ﬁstula time (day)**	3.14 ± 2.54	4.82 ± 3.27	−2.960	**0**.**004**
**Indwelling catheter time (day)**	2.37 ± 1.13	4.98 ± 1.47	−10.231	**0**.**000**
Hematuria time (day)	5.50 ± 1.92	5.72 ± 2.37	−0.526	0.600
**Postoperative hospital stays (day)**	6.67 ± 2.09	7.96 ± 2.02	−3.194	**0**.**002**
Total hospital stays (day)	12.75 ± 3.22	13.88 ± 3.28	−1.784	0.077

^a^ Values are expressed as mean ± standard deviation.

^b^ The independent samples t-test or Mann-Whitney test for continuous variables.

VAS, visual analogue scale.

**Table 5:** Postoperative complications.

Parameters	ERAS group (*n* = 56)	Traditional group (n = 50)	χ^2^^a^	*P*
Complication (*n*, %)	12,21.43%	13,26.00%	0.306	0.580
Fever (*n*, %)	7,12.50%	6,12.00%	0.006	0.983
Hydrothorax (*n*, %)	1,1.78%	0	-	-
Prolonged hematuria (*n*, %)	4,7.14%	7,14.00%	1.335	0.248
Transfusion (*n*, %)	3,5.36%	4,8.00%	0.299	0.584
DSA embolization (*n*, %)	1,1.78%	1,2.00%	0.007	0.935
Urosepsis (*n*, %)	1,1.78%	0	-	-

^a^ Chi-square test for categorical variables.

DSA, digital subtraction angiography.

**Table 6:** Stone composition.

Parameters	ERAS group (*n* = 56)	Traditional group (*n* = 50)	χ^2^^a^	*P*
Calcium-based	37	31	0.190	0.663
Uric acid	6	2	1.707	0.191
Struvite	13	16	1.026	0.311
Cystine	0	1	-	-

^a^ Chi-square test for categorical variables.

## 4. Discussion

ERAS is a program with a series of standardized protocols developed by multi-department collaboration. It aims to improve the outcomes of patient recovery after surgery by minimizing the negative stress effects of surgery, effective analgesia, early mobilization, and early nutrition intake (33). In the past few years, the ERAS program has gradually been applied in the management of prostate cancer and bladder tumor by urologists (29, 34, 35). However, few studies have reported the implementation of ERAS in PCNL for treating staghorn calculi. Besides, the current ERAS program for surgery pays much attention to multi-department collaboration, without much focus on patient participation. This study explored a patient-centered ERAS in PCNL for staghorn calculi based on existing protocols suited for the actual conditions. Patient-centered care is the core concept, and the key principle is attention to patient participation during the whole treatment process, ranging from medical decision making to follow-up. In the ERAS program, the main method is full elimination of the information barrier on diseases and therapies between patients and medical staff through adequate education and communication using various approaches, such as multimedia.

Patient education is an integral part of the ERAS program, especially in patient-centered ERAS, and encompasses the whole process of diagnosis and treatment. The formation and recurrence of urinary calculi have a chronic pathologic basis and are closely related to nutrient intake (11, 29, 36). The contents of our patient education in the ERAS group included not only the ERAS concept, advantages and disadvantages of PCNL, perioperative complications and corresponding treatment measures, and importance of cooperation but also the relationship between the formation and recurrence of urinary calculi and nutrition. Additionally, the importance of nutritional therapy outside the hospital based on different stone compositions in preventing stone recurrence was emphasized repeatedly in every follow-up session by call or WeChat. Only 30.2% of patients were found to be adherent to the strict and complex nutritional therapies 6 months after surgery (36). Therefore, this study simplified the nutritional therapies and performed a strict supervision during every follow-up session to improve adherence and participation of patients on nutritional therapy (Table 7). Beyond these interventions, we showed patients the endoscopy video of a successful lithotripsy procedure to eliminate their preoperative tension and improve their confidence in the surgery. Subsequently, we improved the training exercises of patients involved in abdominal breathing, respiratory frequency regulation, prone positioning above a cushion, and lower limb stretching on the bed in preparation for early ambulation.

**Table 7:** Nutritional recommendations summarized for different stone compositions.

Composition	Recommendations	Unsuitable Beverages for all
Calcium based:	Water intake: 2.0–2.5 L/day	Green tea, black tea, and caffeinated coffee (maximum 0.5 L/day) Sugar-sweetened soft drinks, including cola Alcoholic beverages, including wine and beer
	Avoid oxalate-rich foods	
	Calcium intake: 1,000–1,200 mg/day	
	Protein intake: 0.8–1.0 g/kg normal body weight/day	
	Sodium chloride intake: <6 g/day	
	Increased intake of vegetables and fruits	
Uric acid	Water intake: 2.0–2.5 L/day	
	Protein intake: 0.8–1.0g/kg normal body weight/day	
	Reduced dietary purine intake	
	Increased intake of vegetables and fruits	
Struvite	Water intake: 2.0–2.5 L/day	
	Protein intake: 0.8–1.0g/kg normal body weight/day	
	Sodium chloride intake: <6 g/day	
	Increased intake of vegetables and fruits	
Cystine	Water intake: at least 3.0 L/day	
	Protein intake: 0.8–1.0g/kg normal body weight/day	
	Sodium chloride intake: <6 g/day	
	Increased intake of vegetables and fruits	

Nutrition is essential for postoperative recovery of patients who have undergone surgery. Preoperative nutritional inadequacy is often an important cause of postoperative metabolic stress and insulin resistance, which often lead to weakness and increased mortality in severe cases (37). PCNL is a major surgery in urology. Therefore, nutritional management, which involves interdisciplinary collaboration, was a key component of ERAS in PCNL for staghorn calculi. Excessive intraoperative or postoperative fluid infusion can cause several complications, such as intestinal edema, hypervolemia, coagulation dysfunction, and wound healing delays; whereas restricted fluid intake can relieve pain and quicken recovery (38). Hence, nutritionists are consulted to assess patients' nutritional conditions with the aim of forming a personalized nutrition plan after hospitalization. In the ERAS group, 400 ml of 5% glucose drinks was administered 2 h before surgery, and intraoperative restricted fluid infusion with a standard of 10–15 ml/kg body weight was administered. Early oral intake can increase gastrointestinal activity, promote bowel movement, facilitate higher cognitive function, reduce infectious complications, and improve patient satisfaction (38, 39). Therefore, patients in the ERAS group consumed liquid diets 6 h after surgery and chewed gum.

Preoperative fasting and intestinal preparation are important components of the nutritional plan. Traditional preoperative fasting and intestinal preparation for PCNL are mainly used to prevent anesthetic aspiration and reduce postoperative infection. In fact, enema and long-term fasting before surgery often cause complications, such as pain, bleeding, infection, intestinal mucosal architectural change, electrolyte disturbance, and dehydration (40, 41). In the ERAS group, no preoperative intestinal preparation and routine fasting was performed. No anesthetic aspiration occurred during surgery.

The maintenance of normal body temperature is necessary for life (42). The incidence of hypothermia of varied degrees, which most surgical patients experience during the perioperative period, is 4%–90% (43). Additionally, routine fluid intake during surgery and endoscopic procedures, such as PCNL, require continuous irrigation of fluids for better visualization and removal of stones and blood clots. This often causes a drop in body temperature (44). Perioperative hypothermia is closely associated with multiple complications, including surgical site infection, coagulopathy, slow drug metabolism, increased cardiovascular events, and prolonged hospital stay (45). The maintenance of perioperative body temperature is a core component of clinical pathways in the ERAS program (46). Therefore, body temperature was strictly monitored, the operating room temperature was kept as 24–26°C, and intravenous fluids and surgical infusion fluids were warmed when lithotripsy was performed in the ERAS group to reduce postoperative complications.

Analgesia is an important part of the ERAS program. Few studies reported that the incidence of moderate-to-severe pain after PCNL was approximately 60%, which affected patients, regarding postoperative rehabilitation, daily activities, quality of life, and social and economic conditions (47, 48). Opioids used in traditional analgesia have side effects, such as nausea, vomiting, and intestinal ileus. Therefore, nonsteroidal anti-inflammatory drugs (NSAID), such as parecoxib and flurbiprofen, are recommended (48). Preemptive analgesia during operation, multimodal analgesia based on NSAIDs, and personalized administration based on VAS scoring were performed in the ERAS group.

Adequate analgesia can contribute to early mobilization and diminish postoperative stress response (48). Early mobilization has positive effects on muscle strength, bowel function, cardiovascular and respiratory functions, psychological well-being, and venous thromboembolism prevention (39). Hence, patients in the ERAS group underwent training exercises before surgery. They were encouraged to perform bed exercises, such as lower limb exercises aided by caregivers, which gradually transit to voluntary activities 2 h after surgery. In this study, the VAS 6 h after surgery was lower in the ERAS group (1.32 ± 0.66) than in the traditional group (2.24 ± 0.82) (*P* < 0.05). The ambulation off bed time (1.64 ± 0.61 days) in the ERAS group was lower than that in the traditional group (2.86 ± 0.67 days) (*P* < 0.05). These findings showed a trend of postoperative pain relief and early ambulation off bed in the ERAS group.

Indwelling nephrostomy after PCNL was supposed to press the puncture tract, strengthen drainage and hemostasis, reduce urine extravasation, lower the risk of infection, and improve convenience of the secondary procedure in the traditional concept (49). According to a recent study, early removal of the nephrostomy tube or tubeless nephrostomy does not increase the risk of bleeding, infection, and urine extravasation (50). Due to the high probability of a secondary session in PCNL for staghorn calculi, all patients underwent indwelled nephrostomy after surgery in this study. Additionally, early removal of nephrostomy was encouraged in the ERAS group. No difference in decrease of hemoglobin, hematuria time, and complications were recorded (*P* > 0.05). The indwelling ﬁstula time (3.14 ± 2.54 days), indwelling catheter time (2.37 ± 1.13 days), postoperative hospital stay (6.67 ± 2.09 days) in the EARS group were lower than those (4.82 ± 3.27 days, 4.98 ± 1.47 days, and 7.96 ± 2.02 days) in the traditional group (*P* < 0.05). In the ERAS group, the outcomes showed a trend of decrease in discomfort, acceleration to early exercise, and reduction of postoperative hospitalization time.

PCNL of staghorn calculi often requires multiple procedures to achieve a satisfactory stone clearance rate (6, 7). However, multiple sessions of PCNL increase costs and risk of complications. The stone burden, stone CT value, tract size, and tract number may be the contributory factors. In this study, no differences in stone burden, stone CT value, tract size, and tract number between both groups were recorded (*P* > 0.05). The multiple session rate in the ERAS group (19, 28.57%) was lower than that in the traditional group (30, 60%, *P* = 0.007). Three possible explanations were considered. First, a combination of pneumatic and holmium laser lithotripsy was used in PCNL for staghorn calculi. The former has been shown to have a higher efficiency in the removal of staghorn calculi (51). Therefore, adequate preoperative nutritional support allowed patients in the ERAS group to tolerate longer periods of pneumatic lithotripsy. Second, after preoperative education and communication, the patients could coordinate with the surgeon by voluntary regulation of the respiratory frequency during the lithotripsy procedure. Third, a higher CT value was associated with harder stones (52). The CT value in the ERAS group (975.41 ± 242.17) was lower than that in the traditional group (1,055.66 ± 175.85) (Although *P* = 0.052). This may affect the efficiency of lithotripsy to an extent.

Nutritional intake after discharge is closely related to the recurrence rate of kidney stones (11, 29). The 5-year recurrence rate of calculi ranges from 31.5% to 50%, whereas the 20-year recurrence rate is as high as 75% (53, 54). The 1-year recurrence rate (7, 17.5%) in the ERAS group was lower than that in the traditional group (14, 38.9%) (*P* = 0.037). The monthly follow-up, which encouraged patients to actively or passively adhere to nutritional recommendations, could account for these findings.

The study had few limitations. First, this study was a single-center retrospective study. Second, the study used a small sample size. Third, the follow-up period was short-term; it was 1 year post-surgery. Further multi-center studies with a robust design, adequate sample size, and long follow-up period are required to confirm the conclusions and develop the ERAS protocols in PCNL for staghorn calculi.

## 5. Conclusion

The patient-centered ERAS in PCNL for staghorn calculi accelerated rehabilitation by relieving postoperative pain, shortening hospitalization time, accelerating early ambulation, and reducing multiple session rate and 1-year stone recurrence rate, which have socioeconomic benefits.

## Data Availability

The original contributions presented in the study are included in the article/Supplementary Material, further inquiries can be directed to the corresponding author/s.
